# Abstracts and Gleanings

**Published:** 1888-05

**Authors:** 


### ABSTRACTS AND GLEANINGS.

**Asclepias Syriaca (Milkweed) for Lumbago and Dropsy.** —It is indigenous in Western Missouri, and very generally, I believe, in the Middle and Western States. It grows on rich land, in fence corners, meadows and cleared lands not used as pastures. It is perennial; grows rapidly from early spring until August, when it is from three to six feet high. It has a purple stock as thick as a lead pencil, or even the size of an index finger. It has thick fleshy leaves, very brittle, and exuding a thick, gummy, milky juice; it generally has many branches at top; blooms in August; flowers in bunches, and is purple in color; bears a rough pod as long as a finger, and from half an inch to an inch in diameter at base, tapering to a point thorn-shaped, filled with a white, fibrous, silky furze, in which are the seeds. The root grows perpendicularly, a foot to eighteen inches in length, almost without rootlets, is white and tough; the root is the part used, and may be dug any time from August until April. Its physiological action seems to be diuretic, and mildly laxative. It increases digestion, and stimulates the appetite. That is as far as I have observed its action. I believe it is to some extent a heart tonic, though of that I am not positive as yet.

In 1873, I had a severe lumbago, for which I had tried various remedies, both internal and local, including electricity; but without relief. An old farmer said he could cure me, and related his own experience, and furnished the roots. I began using a decoction, drinking a half teacupful four or five times daily, and in a few days was completely relieved. Then I recommended it to all my patrons who called for treatment, and heard from them the most extravagant praises of its virtues, in return for my suggestions. Amongst my first cases was a man about fifty-eight years old, a painter, who had been disabled from work for nearly a month. He said that when he attempted to arise of mornings that he had to roll out of bed on to the floor, and hold to the bed-post with one hand while attempting to pull on his pantaloons with the other. He could not stand still, but had to keep moving constantly until he could get a seat. I gave him some of the roots, and he used the tea through the day and next morning, got out of bed and dressed with but little pain, and in a week was able to resume his trade. Since then I have met and treated perhaps a hundred cases with like results.

In 1875 or 1876, I saw an article in the *Medical and Surgical Reporter*, from some physician in New York (his name and location I have forgotten), stating the action of *asclepias syriaca* in dropsy. At that time I had a typical case of general anasarca, in a man sixty-five years old. He had heart disease badly. His case grew from bad to worse, baffling me with the best remedies I could find. The anasarca had extended up to the thorax He could not lie down. His body and limbs were distended until the skin seemed ready to burst. I regarded his case hopeless, but concluded to give the silkweed a trial. I punctured his limbs in numerous places and wrapped them in cloths, and thus started a flow of the water. I put him on a free use of the tea, and in ten days he was reduced to a mere skeleton; but could lie down and sleep comfortably. His appetite returned; his bowels from being torpid, acted normally, and there was constant healthy action of the kidneys. His heart's action became more regular and its impulse strong. He lived a year and a half happy and content, when his heart suddenly failed, and he died in a few hours. I have used the same remedy in a few similar cases since, and it has proved the *best* of all remedies so far.—*Medical and Surgical Reporter.*

**Two Cases of Placenta Prævia.**—CASE I.—Mrs. B., æt. about thirty; second pregnancy; was called in consultation with another physician on evening of October 1, 1887. Learned from attending physician that the patient had been suffering from uterine hemorrhage for several weeks, more or less every few days. Found os dilated to about the size of a silver dollar. The hæmorrhage continued despite the tamponing with sponges and strips of cloth. After waiting about an hour and finding the os sufficiently enlarged, I inserted the hand, detaching the placenta upon one side, turned the child and delivered, leaving the head lodged in the pelvis twenty minutes, to check hemorrhage as much as possible. The placenta came away with the fœtus. The patient continued to sink, and died two hours after delivery. Pregnancy was seven month advanced. Delivered under chloroform anæsthesia.

CASA II.—Mrs. W., æt. thirty-five years; third pregnancy; was called morning of February 11, 1888, to find that patient had lost a pint of blood in the two to three minutes that the hæmorrhage continued. The husband had tamponed with a silk handkerchief saturated with sweet oil. Thinking it not best to disturb the patient, left some powdered per-sulphate of iron to be used on a second tampon if necessary. I informed them if anything like this last hemorrhage should occur again, an immediate delivery would be necessary. Thirty hours afterwards I removed the tampon without further loss of blood and could detect, upon examination, the placenta covering the entire region of the cervix. Was called again February 15, about midnight and found the flow more severe than before. The husband failing to tampon successfully, caught the patient by the feet and was holding her head downward. Allowing her to resume the recumbent position, inserted my hand into the vagina and found the os sufficiently dilated to crowd the thumb and index finger into the opening, and by crossing and flexing them form a perfect tampon.

This method of tamponing served two other purposes. By adding the other fingers, one at a time, in half an hour I was able to pass the hand through the os, efficient labor pains coming on in the meantime, having been entirely absent before. Now, making a rent in the placenta near its center I turned the fœtus (a six months') and delivered as in the former case. Immediately tamponed with strips of cloth covered with Monsel's powder, and was gratified to see the case proceed favorably.

Eighteen hours after, upon disturbing the tampon for the purpose of catheterization. I was surprised to see the blood start freely. Applied more per-sulphate and thirty-six hours after de-delivery removed the tampon, and twelve hours later began using vaginal wash. Delivered without anæsthesia. The patient at this writing, March 3rd 1888, has nearly recovered. She had suffered from hæmorrhage since the fourth month, but I did not see her as she was visiting somewhere in Canada.—*Dr. A. J. Collar, in Medical Age.*

**Concerning Chloroform**—Hunter McGuire is reported as saying: "There is one hazard from chloroform we are apt to forget—operating during partial anæsthesia. Many of the deaths from cloroform have happened this way. A tooth is to be drawn, a pile tied, a felon opened, or some operation performed which is the work of a few seconds; and the inclination is to operate before the anæsthesia is complete. It is extremely hazardous to do this. Possibly the brain has not lost consciousness, and the patient is dimly aware that the operation has begun, that the pain he dreads is to follow, and in consequence of his terror fatal syncope comes on. Or, if intellectual consciousness is lost, there seems to be—if I may so speak—a consciousness still left in the nerve centres which preside over the heart and circulation. and the impression of pain on them is fatal. Ether is safer when the operation is to be performed with partial anæsthesia. Of all the elements of danger from chloroform, fear on the part of the patient is the greatest. If the anæsthetic causes real alarm, the heart is nervously weak and the hazard to life especially great. All things being equal, ether, then, is the safer agent. To the dread of the operation the added dread of the anæsthetic produces an emotional condition of absolute terror. Fatal cases under such circumstances are not uncommon. It is safer, if words and a calm manner fail to quiet the patient and allay all anxiety, to give hypodermically one-fourth grain of morphia and one-hundredth grain of atropia, and wait fifteen or twenty minutes for the physiological effects of these drugs before giving the anæsthetic. Emotional excitement increases the chances of paralysis of the nerve centres which preside over the circulation. Morphia obtunds the sensibility of the nervous system, and at the same time is a cardiac stimulant. Atropia is still more powerful in this direction, and the employment of these drugs under such circumstances will lessen or completely remove the danger.

Children take chloroform safely and well. The principal reason for this is that they are ignorant of its danger and are not afraid of being killed by it. How else can we explain the comparative immunity from danger to the young?—*Post-Graduate.*

**Whoop ng Cough.**—Antipyrine in doses of 1 to 2 grains every two hours to a child of five years is the most successful treatment for whooping cough yet discovered.—*Texas Courier-Record.*

**A Case of Profound Opium Poisoning Cured with Amyl Nitrite.**—Dr. McNeill called at my office and requested me to visit with him a woman said to have taken 2½ ounces of laudanum three hours since.

From the appearance of the woman when he left her (only ten or fifteen minutes before coming to my office), he thought she might already have passed away, as she was then apparently in a dying condition.

We found a middle aged woman, over a bar-room, lying on a bed, totally unconscious; face and hands of a livid purple; pulse extremely feeble, rapid, flickering, intermitting; pupils dilated, but ragged in outline, like the pupils of a dead person; respiration did not exceed four per minute—in truth, I did not suppose she could possibly live fifteen minutes.

Having seen in some Western journal the report of a case of opium poisoning in which amyl nitrite had been successfully used, I had requested Dr. McNeill, on my way to the house, to procure some pearls from the drug store. A hypodermic of atropin had been given at the doctor's first visit, but with no visible effect.

The first pearl was broken and imperfectly administered. The second was more carefully prepared, and the effect was most wonderful, calling forth expressions of surprise from those looking on. The face and hands quickly resumed their natural hue. The respiration gradually, but rapidly, became natural and the pulse became more steady, though still rapid and weak. Speaking to her, she opened her eyes, aroused thoroughly to her surroundings and requested (not very politely) to be left alone.

From this time nothing more was required, and I lost sight of her. She had been drinking, but she had undoubtedly taken the laudanum, of full officinal strength, and she certainly would have died but for the use of the amyl nitrite.

I had almost neglected to state that *one* pupil remained dilated for some days afterwards.

Of great service in angina pectoris, used beneficially in asthma, and also to counteract the lethal effects of chloroform, and now arousing to action the almost extinct vital powers of the system of the nearly fatal effects of opium poisoning, it certainly deserves a fair trial at the hands of the profession in all cases of like nature.—*Dr. Haigh, in North Carolia Medical Journal.*

**Fistula in Ano.**—The following method is suggested by Dr. Southworth (*Medical Brief*, April 1888): I take a two ounce bottle cork, make two holes through it lengthwise, pass through a silk ligature, common size, tie one end and wax well; scrape free end and wind it on a flexible probe close below the bulb; now grease the anus and fingers well and insert probe in fistula as far as the opening in the gut. Then introduce the finger, and when the opening is found bend it down and out, take off the string, put your finger up and bend and draw it back without pain. Pass the free end through the cork and tie, not too tight at first, and tighten gently every second day, so as not to cause pain. I have never treated one in this manner but that the cure was complete.

If you have any doubts you can inject into the fistula (if the complete solution will pass into the gut) with the following wash:

℞. Zinci sulph ................................ ʒ ss,

Sodæ carb ................................ ʒ j,

Aquæ ................................ pint j.

M. Sig.: Inject three times a day, and after every discharge of the bowels.

Give tonics and alteratives as indicated. When ligature cuts out use the following ointment for one week, applied twice a day. I give this ointment again as there are always some new subscribers to the *Brief*, yet I think all practitioners should take it as it is our greatest source of knowledge:

℞. Crocus martis ................................ ʒ j,

Zinci ................................ ʒ ss,

Butyrum (Fresh) ................................ ℥ iv.

M. Sig.: Apply as directed above.

One doctor could not get the crocus martis. It is made by taking a piece of sheet iron, ten inches square (or any size wanted), bend it up and put a pound or more sulphate of iron on it, then put it in the stove and heat it until it is red hot. When cool, pulverize and sift. Keep in corked vials.

**New Remedy for Cystitis** —(*California Medical Journal*) Having seen nothing concerning the new remedy for cystitis and hyperesthesia of the genito-urinary tract, pichi (Fabiana imbricata), and being very much pleased with it, I will report, briefly, its action in a few cases. The first case was one of cancer of the uterus, where the whole anterior part of the vagina was indurated and contracted—the patient having to urinate every half hour all night, and the pain would start the tears every time. I gave the following prescription:

℞. Extract pichi ................................ ʒ v,

Liqur potass ................................ ʒ ss,.

Elixir aromat ................................ q. s. ad ℥ iij.

M. Sig.—A teaspoonful every three hours.

In less than two days—in fact, the first night—she had to get up but once She took the medicine irregularly, as required, until she returned home, which was three weeks after, and it controlled the painful urination completely. Neither did she have the backache, which has been a constant accompaniment heretofore.

CASE II.—A lady, with painful and frequent urination, having to get up four times a night. She had been over-treated by one of the too numerous class who see a cause for every ill that woman is heir to through a vaginal speculum. In this case the remedy acted promptly, remedying the backache as well.

CASE III.—A man with a *mild* gonorrhœa. Stopped all scalding of the urine at once.

CASE IV.—An old lady, aged eighty-three, who said it appeared very strange none of the doctors could do her any good. She had to get up several times at night to urinate. She was all right in twenty-four hours, and has continued so since.

I have tried local applications in two cases of vaginitis, and they were greatly benefitted, and ceased using it. Am now anxiously watching for an old man, with prostatitis and cystitis, to come along.

I believe P., D. & Co. alone handle this drug now.

Have considerably lessened the first named dose, now give 10 drops once in three hours.—*Peoria Medical Monthly.*

**The Mutual Relation between the Saliva and the Gastric Juice.**—George Sticker (Volkmann's *Sammlung kl. Vortrage*, No. 297) has made some investigations on the action of saliva which tend to alter materially our ideas of stomach digestion.

The view thus far held has been that the saliva exerts its amylolytic action until the increasing secretion of acid gastric juice checks it, and that then the peptonizing of albuminoids begins. Sticker has come to opposite conclusions.

According to him, the importance of saliva lies in the fact that absence of it causes a decrease or total failure of secretion of gastric juice, and that want of saliva causes not only abolition of amylolytic action, but also influences essentially the gastric proteolysis.

The basis of this investigation was furnished by a patient who had no salivary secretion. Examination of the gastric contents showed not only diminution of amyloid, but also of proteid, digestion, consequently diminished secretion of gastric juice. Administration of jaborandi not only re-established the saliva, but caused a return of the normal gastric secretion, so that in two weeks the patient had entirely recovered.

Since the jaborandi could not have affected the stomach directly, it may be assumed that the secretion of saliva, and its introduction into the stomach, excited the latter to activity.

This view is confirmed by an experiment made by Sticker to that end. Egg-albumin and starch were introduced into the stomach and allowed to remain two hours, in one case avoiding swallowing saliva, in another allowing it to mix in the normal manner with the contents of the stomach.

In the latter case the stomach was found empty, in the former undigested starch and albumen were found and the gastric juice possessed but slight peptonizing power.—*Fortschritte der Medican.—American Journal Medical Science.*

**Abating Typhoid Fever.**—Dr. Forcheimer, in *American Lancet* says: I believe in the p sibility of aborting typhoid fever with calomel. If I get a case before the fifth or sixth day I always give a dose of calomel, and a large one—in some instances repeating it. I then follow this up with rat er full doses of antipyrine, because it lessens pain and seems to have an antiseptic effect. I consider it a very great importance to have two beds, one for the day and one for the night. I choose the best room in the house for the sick room, having no hesitancy in taking the parlor, first removing all the bric-a-brac. The room should be one well lighted and well ventilated, and near the water conveniences. The diet should be absolutely fluid. It is not so particular that it be albuminous but absolutely fluid, no bread no toast. I have seen hemorrhage caused by bread. The patient will often beg for something solid to eat. We can give them tolu to chew, as this is what they miss in the fluid food, the customary chewing. I frequently give:

Acidi hydrochlorici dil, 1.00,

Syrupi of raspberry, 15.00,

Aquæ, 45.00.

M. S. Take one teaspoonful every hour or two.

I give sustaining measures, mainly whisky. In antipyretics we want to avoid every thing that will cause a collapse. I never give cold baths to children on account of collapse. I give the luke warm bath or cold wrap, and prefer the former.

**Hydrocele.**—A Texas physician, in *Courier-Record*, thus describes Dr. Wyeth's "Radical Cure for Hydrocele." First, make the scrotum strictly aseptic in the fullest sense of the term, then anesthetize the scrotal wall at the most dependent point of the hydrocele by a hypodermic injection of a few minims of four per cent. cocaine solution Now introduce a small trocar and canula or a large sized hypodermic needle, and allow the fluid of the hydrocele to run out—not, however, *squeezing* it out, as the intention is to leave about a dram of fluid in the sac. Next inject from 5 to 30 minims of pure carbolic acid into the sac, through the canula or needle, withdraw the latter, and get the acid well in contact with the entire sac. Now, your patient is ready to resume his duties, you leaving the acid in the sac, except it be the largest amount used; and as to the amount of acid used, that depends altogether on the size of the sac; hydroceles holding a quart of fluid requiring as much as 30 minims, while the smaller ones require less, as low as 5 minims.

The operation is perfectly simple, safe and painless, and not a single case has had recurrence of the trouble where this was used; moreover the patient does not quit work even for one hour, much less is he required to go to bed. This treatment of hydrocele may well be called "Wyeth's."

**Hydrate of Amylene.**—Professor Mirny has employed the hydrate of amylene ($C_2 H_{12} O$) for two years. The experiments on animals show that the medicine has no disadvantageous action on respiration or circulation, but produces a profound sleep. In average doses it agitates the encephalon, in stronger doses the cord and medulla, the reflexes disappear, the respiration is arrested, afterwards the heart. It is a better hypnotic than paraldehyde, and more agreeable to take. It does not exercise such influence on the cardiac activity as chloral, which lowers arterial tension.

Hydrate of amylene administered by mouth or lavement, in doses of 3 to 5 grammes, produces in about half an hour a calm sleep of from six to twelve hours duration. There is no period of excitement, no nausea or cephalalgia, and no congestive phenomena. But it is necessary to have a preparation absolutely pure. In cases of insomnia during pain. such as sciatica, etc., it is better to associate the amylene with morphine. One gramme of chloral, 2 grammes of hydrate of amylene, or 3 grammes of paraldehyde have the same action as an hypnotic. It is employed with success at the Clinique of Mental Maladies of Professor Joly, of Strasburg.—*Medical Times.*

**Apomorphia.**—This alkaloid, prepared from morphine, is a snow-white powder, is permanent when dry, but becomes green when moist. A solution turns green in a few hours, and finally black. This change of color is not attended by loss of its specific drug action. It can be prepared in triturations or in alcohol. The hydrochlorate is generally preferred. This drug seems to contain all the emetic powers of opium and its alkaloids. As small a quantity as 1-20 grain injected under the skin, or taken into the stomach of an adult, will cause *sudden, painless vomiting* in five or ten minutes. Its method of action (if Dr. E. M Hale is correct) would narrow its therapeutic sphere to those cases o vomiting due to general irritation, or from purely nervous (central) causes —possibly from remote reflex irritation It does not seem to derange the functions of the stomach for any length of time, as do other emetics.

One of the most useful properties of this drug is its power to cause sudden and complete emesis in case of poisoning, or to expel fermenting or noxious substances from the stomach. For this purpose inject hypodermatically, or give internally one-fifth or one-fourth of a grain. If it causes too great depression, give 1-100 grain of glonoin, which will quickly restore the patient.—*Medical Times.*

**How to Grow Fat.**—The *Medical Review* says that among some of the directions given to thin people with the hope of making them fat are the following:

They must sleep all they can; keep early hours for retiring; lie down in the middle of the day; drink a great deal of water; eat heartily, especially of farinaceous food; take plenty of exercise, but in moderation. Be cheerful. Sterne says that "every time a man laughs he adds something to his life." And according to Solomon, "A merry heart doth good like medicine, but a broken spirit drieth the bones." Follow the old adage: "Laugh and grow fat."—*Pacific Record.*

**Salicylate of Soda** has a certain superiority to other means in severe, generalized, articular rheumatism accompanied with fever; but if the heart is weak, if there has been any previous debility or trouble in compensation as the result of old valvular lesions, it is best to use antipyrin. Salicylate of soda is a heart depressant, while antipyrin never produces the least perturbation in the contractile power of the cardiac musculature, and possesses, therefore, great advantages in the conditions just described. Further than this, antipyrin, on account of its harmlessness and certainty of action is better than salicylate of soda in rheumatism where there is no fever. Antifebrin in this disease has no advantages over antipyrin, and salol is inferior to all of them. Antipyrin is of great service in the treatment of hepatic and renal colic, and the author has never seen it fail in the second of these conditions; the pain is relieved, and in twenty-four hours the calculus is expelled. Antipyrin is no less useful in membranous dysmenorrhœa. It may be given hypodermically, by the mouth or by the rectum.—*Union Medicale, Paris.*

**Acid Treatment of Intestinal Diseases of Infancy and Childhood.**—Dr. D. P. McLachlan, of York, Michigan, in a paper read before his County Society, says: Regarding the use of opium, there can be no question. There is no remedy which will so effectually meet the second indication, viz: to allay irritation, check the increased peristaltic action of the bowels, and arrest the excessive secretion of the intestinal follicles. When to opium we have added a remedy which will check fermentation, and one that is at the same time tonic and astringent, we have met every indication in the treatment. In the mineral acids we have just such a remedy as we need, and if the fellows of this society will leave chalk mixture entirely out of their prescriptions and substitute nitro-hydrochloric acid, unless their experience differs very materially from mine, they will be well pleased with the change.

Over seven years ago I became convinced that acids were (theoretically, at least) indicated rather than alkalies, and commenced their use, and since that time I have never given a particle of alkali to a patient of any age as a remedy for diarrhœa.

I have given nitro-hydrochloric acid in combination with paregoric to a child three weeks old with the best results.

A favorite prescription with me for a child one year old is—

℞. Tincturæ opii ................................ gtt. x,

Acid. nitro-muriatic ........................... gtt. v,

Syrup. aurantii corticis, q. s. ad ................ f ℥ ij.

Sig.: Teaspoonful every two or three hours.

I have given this prescription a great many times in the last seven years, and it has never disappointed me.—*C. and C. Record.*

**Tying the Umbilical Cord.**—Dr. Camp (*Mass. Med. Jour.*) uses the following method. He first ties and cuts the cord two inches long, then washes off the cord with equal parts of Listerine, which is without doubt one of the very best antiseptics and deodorizers, and glycerine, retie it within an inch of the naval with a ligature of No. 13 iron dyed surgeon's silk, and cut off the superfluous part of the cord containing the first ligature. Then I wrap the cord in borated absorbent cotton, and apply the belly-band in the usual way. The band must be reapplied occasionally, but the cord is not to be touched again until the sixth or seventh day, when, if it does not come off easily, it is snipped off by the scissors. If this article will induce the reader to try this method in one case, he will not go back to burnt muslin, linen with a hole in it, etc.

**How to Treat Cramps in the Leg.**—There is nothing easier than to make the spasm let go its hold, and it can be accomplished without sending for a doctor, who may be tired and in need of a good night's rest. When I have a patient who is subject to cramp, I always advise him to provide himself with a good strong cord. A long garter will do if nothing else is handy. When the cramp comes on, take the cord, wind it around the leg. over the place that is cramped, and take an end in each hand and give it a sharp pull—one that will hurt a little. Instantly the cramp will let up, and the sufferer can go to bed assured it will not come on again that night. For the permanent cure, give about 6 or 8 cells of galvanic battery, with the negative pole applied over the spot that cramps, and the positive pole over the thigh. Give it for ten minutes, and repeat every week for a month.

I have saved myself many a good night's rest, simply by posting my patients, subject to spasm of the legs, how to use the cord as above. I have never known it to fail, and I have tried it after they had worked half the night, and the patient was in the most intense agony. Even in such cases, at the first jerk of the cord, all pain left.—*Dr. R. W. St. Clair, in Medical Age.*

**Antipyrin as Homestatic and Disinfectant.**—Antipyrin seems to extend its uses every day. Dr. Henocque tells us of its hemostatic and disinfectant properties. For the first it may be employed in powder or in the form of a pomade, or, again, in a solution of one to twenty for washing wounds. Epistaxis is stopped by blowing powdered antipyrin into the note. The drug is also being incorporated into cotton and paper for dressings. In ulcerated cancer of the breast, for instance, a mixtnre of one part of antipyrin to three parts of vaseline will form a paste that spreads over the ulcerations and covered with wadding (to be renewed twice a week), will prevent bad order and hemorrhages, so that the action is favorable to cicatrization,—*Medical Times*, February 15 h.

**A Successful Treatment of Hydrophobia.**—Dr. Kinchensky, in an elaborate study. from the clinical side, of 693 cases occuring in the hospital at Moscow, reports a case in which success followed a peculiar treatment (quoted in the *Bull. Gin. de Therepuetique*, January 15, 1888.)

A woman. aged thirty-five, was bitten by a mad dog five weeks before her admission to the hospital. She presented all of the usual symptoms of the disease in their most typical form. She was placed in a warm bath, where she was bled to syncope, losing fourteen ounces. During the two following days she was given ten grammes (160 grains) of powdered belladonna leaves. On the the third day the hydrophobia had disappeared.—*American Journal Medical Science.*

**Ague**—*Treatment.*—If tongue is coated and bowels constipated (as is generally the rule) I give a purgative composed of calomel, rhubarb, ipecac and podophyllin, and five hours before the expected chill I give from 15 to 20 grains of quinine and order patient to remain in bed until after chill time.

In the majority of cases you will not have to give more than two or three doses of quinine if you give the above purgative first.

The above treatment is for acute cases, chronic cases require more physic—as quinine, arsenic, iron and strychnia. A good prescription is one which I saw in the *Brief* last year.

℞. Ol. sassafras..........................gtts. xxx,
Pip. nig..................................gtts. xxx,
Acid. Arsen..............................grs. j,
Strychniæ sulph.........................gr. j,
Quiniæ sulph.............................ʒj.

Make into pills No. 30. Sig.: Take one thrice daily after meals. —*Medical Brief.*

**Embalming Fluid.**—Dr. J. L T. Davidson, Denver, Colorado, *Pharmaceutical Era.*

℞. Commercial white arsenic ................1 lb. av,
Commercial caustic soda (concentrated lye)..6½ oz. av.
Water sufficient..............................

Dissolve the arsenic and the soda in a pint of water by aid of heat, add water to make four pints and filter. Dilute this solution for use with three times its volume of water. One to two gallons of the dilute solution suffice to preserve a body satisfactorily. It should be injected into the femoral artery, except in the cases of cadavers for dissection, when the carotid is generally selected.

A strong solution of zinc chloride, made by dissolving scrap zinc in commercial hydrochloric acid to saturation, may also be employed. The solution (one to two gallons) should be only gradually injected, several hours being occupied in the process to obtain a perfect result.

**Treatment of Ulcers of the Leg.**—The majority of chronic ulcers of the leg do not heal because of the low vitality of the part; the legs are cold and circulation sluggish. The best treatment for these cases is as follows: pour water as hot as the patient can bear it, over the leg from the knee to the foot until the leg feels hot to the hand; take a powder (oxide zinc, 1 part; corn starch, 4 parts) and dust over the surface of the ulcers so long as any will adhere. Envelop the leg in a thin layer of cotton batting and hold this in place by a few turns of a roller bandage. In dressing the leg first remove the bandage, and then pour the hot water over the cotton till it is thoroughly saturated, when it can be readily taken off. The treatment described should be repeated daily. The hot water acts as a stimulant, and tends to induce permanent vitality.—*Weekly Medical Review.*

**Red Hawthorn in Uterine Hemorrhage.**—The root of the *Cratœgus Officinalis* or red hawthorne (Russian, krasny boiaryshnik), has been from time immemorial used by the Russian peasantry as an excellent remedy for uterine hemorrhage of all kinds. With the view of testing its value, Dr E. M. Jdanko, of Piatsgorsk, recently gave (Proceedings of the Russian Balneological Society of Piatsgorsk, August 29th, 1887, p. 35) a very strong decoction of the root to a lady, aged fifty-two, who was suffering from profuse floodings, caused by uterine fibromyoma, for which most of the usual hemostatics had been tried in vain. The use of the hawthorn completely arrested the hemorrhage. Dr. Jdanko therefore suggests that a fair trial should be given to this popular remedy.—*Medical Times.*

**Rapid and Simple Method of Reducing Downward Dislocation of the Shoulder.**—Dr. P. F. Abril fixes the humerus and makes the glenoid cavity descend on to the head of the humerus. He claims for his method that it is most simple easily, and quickly done, that chloroform is not necessary to obtain muscular relaxation, that the pain is trifling, and that no assistants are required. He makes the patient stand with a crutch in his axilla; he then holds the hand of the affected side, making slight traction downwards; the patient is now to let himself down as if he were going to fall on his knees, and as he falls the head of the humerous glides into its normal position, and the patient is surprised at finding himself cured.—*New York Medical Abstract.*

**Erythrophleine.**—Dr. L. Lewin, in a paper read before the Medical Society of Boston, January 11, says of the new alkaloid that a 2 per cent. solution in a dog's eye renders it insensible for from ten to twenty-four hours, and that a solution of 1 20 of 1 per cent. produces anæsthesia of the cornea and conjunctiva continuing from several hours up to two days, gradually decreasing in intensity. The action is altogether local, and if a solution be injected into the eyelid of an animal it becomes so insensible that touch does not induce motion, while the eye itself retains its sensibility. Taken internally its effect upon the heart is somewhat like digitalis. Until more is known of the drug of course it should be used with great caution.—*Medical Times.*

**Remedy for Epithelioma.**—In the *Deutsche Medical Wochenschrift*, Dec. 1, 1887, Dr. Bidder strongly recommends the application to carcinoma of the skin, or uterus, of a powder composed of equal parts of powdered savin and burnt alum· Its application to the skin is madc by rubbing it on the new growth, upon which it will act without effecting the sound tissues. In case of epithelioma of the vaginal portion of the cervix uteri, it can be applied through a speculum and kept in place by a small tampon of cotton or wo l. Used in this way, it lessens hemorrhage, diminishes fever and relieves pain. Dr. Bidder even thinks that it might effect a cure in cases in which the disease has not made much progress.—*Southern Clinic.*

**Permanganate of Potassium in Toothache.**—The following formula has been suggested by Dr. Popoff:

R. Potass. permang. . . . . . . . . . . . . . . . . . . . . . . . . . . .grs. iij,
Aq. destil. or fontanæ. . . . . . . . . . . . . . . . . 1 (Russ.) fl pound.

Misc. One tablespoonful to be taken in the mouth, every half hour, and to be held therein on the affected side for several minutes.

The most agonizing pain is said gradually to disappear in a few hours.—*Russ Meditz.*

**Menthol and Mentholeata.**—A solution containing from 2 to 10 grains of menthol to the ounce of water has been recommended in urticaria and pruritis, and repeated applications in many cases effect a cure. Oleic acid has been found by Professor Remington to be a free solvent of menthol and preferable to volatile solvents. Mentholeata can be made by adding to 200 grains of menthol, in a test-tube, half a fluid ounce of oleic acid, and applying gentle heat until a solution is obtained. This preparation will probably be found useful in pruritic skin affections in which a certain degree of absorption is desired.—*Journal of Cut. and Genito-Urinary Diseases.*

**A New Local Anæsthetic.**—Prof. Wilhelm Filehne, of Breslau, has discovered that the union of tropin, a molecule derived from atropia, with benzoic acid, produces a substance which he names *benzoyltropin.* He states that it produces an "exquisite local anæsthetic effect." It has also mydriatic power, paralyzing the accommodation. Filehne states that it is the benzoyl molecule in cocaine which is the active agent. Benzoyltropin is the nearest approach to cocaine. It is much higher in price, and has greater mydriatic power.—*Popular Science News.*

**The Treatment of Post-partum Hemorrhage.**—Dr. Temple, ot Toronto, in a case of post-partum hemorrhage, recently treated, injected hot water into the uterus several times without avail. He emptied the uterus of clots, and exercised manual compression, but unsuccessfully. In the emergency he injected a tumblerful of undiluted brandy within the uterus; prompt and lasting contraction ensued, and cessation of the hemorrhage.—*Canada Lancet*, January 1, 1888.

**Treatment of Ingrowing Toe-nail.**—Patin recommends (*Gaz. des hop.*) for this affection that the nail be thouroughly bathed with water, then dried, and painted with traumaticin (10 parts of gutta-percha to eighty parts of chloroform). Sometimes it is best to dress the toe with diachylon ointment after painting it. Of course as complete rest as possible is to be given to the toe.—*New York Medical Journal.*

**Goitre.**—The fluid extract of bannana root is said to cure the worst forms of goitre.

**An Important Incompatibility.**—The death of a child was caused not long since by ignorance on the part of a physician of the fact that chlorate of potassium and iodide of iron are incompatible. The reaction between these compounds precipitates the iron in the form of sesquioxdide, setting free the whole of the iodine, according to the equation $2FeI^2 XKClO^3 = Fe^2 O^3 XKCl 2I^2$. This incompatibility has been pointed out repeatedly, but is not always borne in mind by the physician or the dispenser.—*Pharmaceutical Era.*

**Antipyrin in Nocturnal Emissions.**—Dr. Thor, of Beucharest, finds antipyrin an excellent substitute for bromide of potash in nocturnal emissions, being free from the objection to which bromide is liable, viz., that of producing acne. He prescribes from 7 to 15 grains in the form of tablets just before going to bed. Again, in cases of the so-called sexual neurasthenia of Beard, the same drug has proved very useful, but larger doses are required. Dr. Thor commences with 15 grains a day, and gradually increases this to 20 grains.—*Lancet*, *February*, *1888.*

**Sozoidol.**—A new dermatological remedy introduced by Trommensdorff, appears in pure white crystals. It combines three of the best parasiticides known, being composed of iodine, carbolic acid and sulphur. It appears to have decided curative properties in eczemata of all descriptions, herpes squamosus and tonsurans, impetigo and ulcers. It is free from odor and is well borne by sound and diseased skin. It is readily soluble in water and alcohol, and can be used in the form of a powder or a lanolin paste.—*Texas Courier Record.*

**Hip Dislocations.**—Dr. Allis has pointed out several valuable points in diagnosticating *injuries of the hip joint.* If the patient can cross the legs, there is dislocation in the hip joint of the limb raised. In dislocation of the head of the femur upon the dorsum of the ilium, when the foot is inverted, the inside rests upon the bed. If dislocation is into the sciatic notch, the femur at right angle with the body, the sole of the foot comes in direct contact with the bed.—*College and Clinical Record.*

**Cotton-seed Oil is Detected** by M. Labiche in mixture with other oils, by adding to a sample of the oil an equal volume of a saturated solution of lead acetate, followed by water of ammonia, and shaking the mixture vigorously. If cotton-seed oil is present, a red or orange red is produced. No color is produced in olive oil, castor oil, cod liver oil, o l sweet almonds, or in melted butter, unless cotton-seed oil is present as an adulterant.—*Pharmaceutical Era.*

**Salol in Rheumatism**—It is said that "In chronic articular rheumatism salol is preferable in every respect, not only avoiding the dangers attending prolonged use of salicylic acid, but offering more positive assurance of cure."

